# A survey of knowledge and attitudes relating to cervical and breast cancer among women in Ethiopia

**DOI:** 10.1186/s12889-018-5958-8

**Published:** 2018-08-29

**Authors:** Bekele Chaka, Abdul-Rauf Sayed, Bridgette Goeieman, Sarah Rayne

**Affiliations:** 1 AMREF Health, Addis Ababa, Ethiopia; 2 School of Public Health, University of Cape Town, Cape Town, South Africa; 3 Right to Care, Johannesburg, South Africa; 4 Department of Surgery, Helen Joseph Hospital, University of the Witwatersrand, 7 York Road, Parktown, Johannesburg, 2193 South Africa

**Keywords:** Breast cancer, Cervical cancer, Knowledge attitudes and beliefs, Ethiopia, Oncology

## Abstract

**Background:**

Breast cancer and cervical cancer are the two leading cancers among women in Ethiopia. This study investigated knowledge and attitudes related to these two types of cancer among women in 4 zones of Ethiopia. This is the first study employing a validated questionnaire to investigate knowledge and attitudes relating to breast and cervical cancer in Ethiopia.

**Methods:**

A community based cross-sectional study was conducted from September to November 2015 in the North Shewa zone (Amhara region), Gamo Gofa zone (Southern Nations, Nationalities and Peoples’ region) and zones 1 and 3 (Afar region) of Ethiopia. A total of 799 women aged 18 years and older participated in the survey. Multiple logistic regression analysis was used to investigate the association of possible predictors with breast and cervical cancer knowledge.

**Results:**

A total of 799 women aged 18 years and older participated in the survey. Of the women interviewed, 63.0% had heard of breast cancer and 42.2% had heard of cervical cancer. Among those who had heard of breast cancer, 21.3% (107/503) had heard of breast cancer screening and 1.4% of women aged 40 years and older had undergone at least one breast screening examination. Fewer than half of the participants provided the correct response to questions related to risk factors for breast and cervical cancer. Among those who had heard of cervical cancer, 41.5% (140/337) had heard of cervical cancer screening and 3.3% had undergone at least one cervical cancer screening examination. Women with primary and higher levels of education were more likely to have heard of breast cancers (OR = 3.0; 95% CI: 2.1–4.2; *p* < 0.001) and cervical cancer (OR = 1.9; 95% CI: 1.4–2.6; *p* < 0.001). From the overall attitude score, the majority of the women were found to have negative attitudes towards breast cancer (67.4%) and cervical cancer (70.6%).

**Conclusions:**

This study found that the overall knowledge of risk factors for breast cancer and cervical cancer among women was low. Lack of cancer awareness, and lack of education in general, are the most potent barriers to access and care, and should be addressed through multi-faceted strategies including peer-education, mass media and other community-based interventions.

## Background

Cancer is one of the leading causes of morbidity and mortality worldwide. According to the most recent estimates reported by the International Agency for Research on Cancer (IARC) there were approximately 14 million new cases of cancer in 2012 [1]. However, there is a noticeable lack of cancer statistics from low and middle-income countries [2]. The burden of cancer is underestimated in the Federal Democratic Republic of Ethiopia as is the case in other low and middle-income countries [3]. The Ethiopian health service is inadequately equipped to manage the cancer prevention and treatment needs of a large and ever-growing population, and much attention is currently directed to communicable diseases such as HIV, malaria and tuberculosis [3].

In 2012, breast cancer and cervical cancer were the two leading cancers among women in Ethiopia, with an age-adjusted incidence rate of 41.8 and 26.4 per 100,000 women, respectively. According to IARC, an estimated 12,956 new cases of breast cancer and 7095 new cases of cervical cancer were reported in 2012. Annual deaths for all cancers combined among women were 28,711 in 2012. The percentage of breast cancer and cervical cancer deaths were 24.7% and 16.5%, respectively [4].

Despite the high morbidity and mortality rates of cervical and breast cancer in Ethiopia, there are very few community-level interventions. A general lack of awareness, ineffective screening programmes, and insufficient attention paid to women’s health, all contribute to alarmingly high cancer rates. A study conducted in three teaching hospitals in Addis Ababa found that only 6.5% of all participants had ever undergone a Pap smear examination for cervical cancer screening [3]. Though there have been a number of studies conducted on the knowledge and attitudes Ethiopians have towards cervical and breast cancer [5–8], no study has employed a standardized questionnaire. The purpose of this study was to describe the knowledge and attitudes of cervical and breast cancer among women aged 18 and above in Ethiopia’s North Shewa zone (Amhara region), Gamo Gofa zone (Southern Nations, Nationalities and Peoples’ region) and zones 1 and 3 (Afar region).

## Methods

A cross-sectional study was conducted from September to November 2015 in the North Shewa zone (Amhara region), Gamo Gofa zone (Southern Nations, Nationalities and Peoples’ region) and zones 1 and 3 (Afar region) of Ethiopia. The study area included 3 regional hospitals, 40 health centers and 200 health posts together serving a population of 2.2 million [9], approximately 24% of the population are women aged 18 and older [10].

### Study setting

Ethiopia is a low-income country in the East of Africa, and although the fastest growing economy in the region, it remains one of the poorest. The government has prepared a national cancer control plan in general, and cervical cancer strategic guidelines and training manual. Routine screening is not available to the population, but some government and private facilities provide mammography and cervical screening, usually in the capital city and region. These are provided only with fee-for-service from the patient, although part of the national plan is to expand these services into other regions of the county in the next five years. Some advocacy and awareness campaigns exist, whether through cancer associations promoting community awareness on the benefit of early screening, or the organization of “commemorate cancer” days in direct association with the government*.* There were not any pre-cervical cancer screening services in the implementing areas during this study, which was a baseline assessment before introduction of screening services (initiation of pre-cervical cancer screening and treatment at health center level; breast self-examination at community level and breast ultrasound examination at hospital level).

### Questionnaire design

There is no validated questionnaire to investigate knowledge and attitudes related to cervical and breast cancer specifically in Ethiopia. For the purpose of this study, questions to determine attitudes were adapted from other validated questionnaires including Champion’s Health Belief Model Scale and Powe’s Fatalism Inventory (modified version) [11–14]. The questionnaire consisted of 8 closed-ended questions that determined knowledge of risk factors and 16 closed-ended questions that determined attitudes related to cervical and breast cancer separately. Questions were chosen based on their relevance to the Ethiopian cultural setting, considering the diversity of cultural and religious beliefs in Ethiopia. The questionnaire was initially developed in English and then translated into the local Amharic language. A pilot study was conducted to test the study instrument and, as a result, some questions were rephrased or omitted to prevent response bias. The final, paper-based questionnaire was structured into the following five sub-sections: i) demographic characteristics; ii) knowledge of cervical cancer; iii) knowledge of breast cancer; iv) attitudes of cervical cancer; and v) attitudes of breast cancer. The structured questionnaire was used by five trained interviewers to interview women aged 18 and older. To increase the reliability of the information, the interviewers were trained to administer the questionnaires in a uniform way to prevent their own interpretation of the questions.

Knowledge scores for the risk factor questions were coded as ‘1’ for a correct response and ‘0’ for an incorrect or ‘not sure’ response. Attitude was assessed on a scale of 1 to 3 (yes/not sure/no, respectively). A negative response was assigned a score of ‘1’; not sure ‘2’; and a positive response ‘3’. An average score was calculated for each participant from the sum total of 16 questions. Those who scored 2.5 and above were categorised as having a ‘positive attitude’, and scores below 2.5 as having a ‘negative attitude’.

### Questionnaire validation

The Kuder-Richardson Formula 20 (KR-20) [15] reliability coefficients and Cronbach’s alpha [16] coefficients were calculated for dichotomously scored variables and variables scored on a scale 1 to 3, respectively. The KR-20 coefficient for the group of questions pertaining to knowledge of risk factors was 0.83 for cervical cancer and 0.62 for breast cancer. Values greater than or equal to 0.70 were considered acceptable [17]. Similarly, the Cronbach’s alpha showed acceptable reliability for the group of questions pertaining to attitude of cervical cancer (∝= 0.75) and breast cancer (∝=0.77).

### Sample size

The sample size was calculated using the formula for estimating a population proportion [18]:

$$ n=\mathrm{P}\left(1-\mathrm{P}\right){(1.96)}^2\div {\mathrm{d}}^2 $$

From our review of the relevant literature [7], we anticipated that the proportion (P) of Ethiopian women with adequate or comprehensive knowledge of female cancers would be about 31% (*P* = 0.31; d = 0.05 is the desired precision). Since this study utilized a multistage cluster sampling method, the sample size was multiplied by the design effect (D = 2), plus a non-response rate of 10%. Therefore, the minimum sample size required for this study was 724. In order to gain sufficient statistical power to explore possible demographic factors that may be associated with level of knowledge of female cancers, a sample of *n* = 800 was used for this study.

### Sampling method

The study participants were selected using a multistage cluster sampling technique. A proportional stratified sample was drawn from each respective regional zone the sample was then allocated among five randomly selected administrative areas called kebeles (the smallest national administrative unit or ward) proportional to the number of households in each kebele. The required number of households from each kebele was selected with a systematic random sampling method, using independent sampling intervals in each kebele. The interviewers were instructed to strictly adhere to the predetermined sampling interval. Only one female participant aged 18 years or older per household was interviewed. When an eligible participant was not available during the first visit, the interviewer arranged alternative visits to complete the data collection.

### Data analysis

Questionnaire data were manually entered into EpiData 3.1 [19] and exported to Stata 13.1 [20] for statistical analysis. Categorical variables are presented as frequency tables, and continuous variables are presented as descriptive measures, expressed as mean and standard deviation or median and range. Odds ratios were used to test the association between the binary variables “ever heard of cervical cancer (yes/no)?” and “ever heard of breast cancer (yes/no)?” and demographic characteristics. Multiple logistic regression analysis was used to investigate the association of possible predictors. For all analyses, a *p*-value of less than 0.05 and a 95% confidence interval that does not span unity were considered as thresholds of statistical significance.

#### Ethical considerations

Ethical clearance for this study was obtained from the respective zonal health departments (Reference number MT1/49/45/488, 3 September 2015). Informed consent forms were signed by the interviewer indicating that the study objectives were explained to the participants and that verbal consent was obtained. Confidentiality was ensured throughout the process of data collection. Analysis was performed using de-identified code numbers rather than participant names.

## Results

From the total sample of 800, 799 women participated in the survey, giving a response rate of 99.9%. The majority of the women interviewed were from the Amhara region (46.3%), followed by the Southern Nations, Nationalities and Peoples’ region (39.3%), and the Afar region (14.4%).

### Socio-demographic characteristics

The median age of the study participants was 30 (range 18 to 69 years) and approximately two-thirds (68.5%) of the participants were aged 20 to 39 years (Table 1). The majority of the participants were married (74.6%) and 61.2% identified as housewives. Twenty-three percent of the participants had a primary level of education while 43.4% were non-literate. Over half (56.6%) of the participants were identified as Orthodox Christians. Study demographics were compared to data from the 2016 Ethiopia Demographic and Health Survey (DHS) report. [21] The distribution by age-group (20–29; 30–39; 40–49) from the DHS report (46.5%; 34.8%; 18.8%) was similar to the study cohort (44.6%; 35.9%; 19.4%). The DHS collected data among 18,008 households at the national level between January and June 2016. The DHS also reported that among women (15 to 49 years of age) 63.9% were married; 35% had attained primary level of education; 47.8% were non-literate and 43.3% identified as Orthodox Christians. A distinction between the DHS cohort and the study population is that our study population lives in predominantly rural areas.

**Table 1:** Socio-demographic characteristics of study participants (*n* = 799)

	Number	Percent
Age		
< 20	51	6.4
20–29	303	37.9
30–39	244	30.5
40–49	132	16.5
50–59	55	6.9
60+	14	1.8
Marital status		
Married	596	74.6
Single	108	13.5
Divorced	64	8.0
Widowed	31	3.9
Occupation		
Housewife	489	61.2
Employed/self-employed	211	26.4
Unemployed	57	7.1
Student	42	5.3
Education		
Non-literate	347	43.4
Primary education	184	23.0
High school	108	13.5
Read and write	107	13.4
Diploma	34	4.3
Technical	15	1.9
University degree	4	0.5
Religion		
Orthodox Christian	452	56.6
Protestant or Catholic	212	26.5
Muslim	134	16.8
Other	1	0.1

### Knowledge of cervical cancer

Fewer than half (42.2%) of the study participants had ever heard of cervical cancer. Among those who had heard of cervical cancer (337/799), 41.5% had heard of cervical cancer screening and only 3.3% had ever undergone a cervical cancer screening examination. Among those who were not willing or reluctant to go for cervical cancer screening (201/799), 89% stated lack of knowledge as the primary reason for not being screened for cervical cancer. Those who had heard of cervical cancer reported that their primary sources of information were from family or friends (57.6%), radio/television (53.7%), health care facility (39.5%), and less than 5% stated social media, newspaper or a non-governmental organization (NGO). Less than half gave the appropriate response to questions related to risk factors for cervical cancer (Table 2). Only 21.4% knew that human papilloma virus (HPV) is a risk factor for cervical cancer.

**Table 2:** Questions related to risk factors for cervical cancer (*n* = 337)

Questions to describe knowledge on risk factors for cervical cancer	Correct responses %
Is cervical cancer preventable?^a^	44.2
Is having many different sexual partners a risk factor?^a^	44.2
Is smoking a risk factor for cervical cancer?^a^	38.6
Is HIV a risk factor for cervical cancer?^a^	26.7
Is oral contraception a risk factor for cervical cancer?^a^	24.6
Is giving birth to many babies a risk factor?^a^	23.7
Is human papilloma virus (HPV) a risk factor for cervical cancer?^a^	21.4
Are you more likely to get cervical cancer if your family has it?^a^	15.7

^a^The correct response for these questions was “Yes”

The multiple logistic regression analysis found a significant association between the outcome variable, “ever heard of cervical cancer (yes/no)?” and demographic variables (age, education, and employment). Women with primary and higher level of education were almost two times more likely to have heard of cervical cancer (OR = 1.9; 95% CI: 1.4–2.6; *p* < 0.001). Employed participants (OR = 1.4; 95% CI: 1.0–2.0; *p* = 0.03) and women who were age 30 or older (OR = 1.4; 95% CI: 1.1–2.0; *p* = 0.023) were more likely to have ever heard of cervical cancer.

### Knowledge of breast cancer

Almost two-thirds (63%) of the women interviewed had ever heard of breast cancer. Among those who had heard of breast cancer (503/799), only 21.3% had heard of breast cancer screening and 1.4% of women aged 40 years and older had ever undergone a breast screening examination. Those who had heard of breast cancer had similar sources of information to the cervical cancer participants, as 63.4% reported that they heard about it from family or friends, radio/television (53.6%), or a health care facility (47.9%). Fewer than one in 20 reported that they heard about it from social media, newspaper or an NGO. Less than half provided the correct response to questions related to risk factors for breast cancer (Table 3).

**Table 3:** Questions related to risk factors for breast cancer (*n* = 503)

Questions to describe knowledge on risk factors for breast cancer	Correct responses %
Do you think bigger breasts are a risk factor for breast cancer?^a^	48.9
Do you think alcohol is a risk factor for breast cancer?^b^	35.4
Do you think breast feeding makes it less likely of getting breast cancer?^b^	22.9
Do you think exercise makes it less likely of getting breast cancer?^b^	21.7
Do you think you are more likely to get breast cancer if someone in your family has it?^b^	19.9
Do you think the oral contraceptive is a risk factor for breast cancer?^b^	15.7
Do you think inherited gene defects can cause breast cancer?^b^	13.7
Do you think a diet with lots of animal meat a risk factor for breast cancer?^b^	9.2

^a^The correct response for this question was “No”; ^b^The correct response for these questions was “Yes”

A multiple logistic regression analysis found a strong association between the outcome variable, “ever heard of breast cancer (yes/no)?” and level of education. Women with primary and higher level of education were three times more likely to have heard of breast cancer (OR = 3.0; 95% CI: 2.1–4.2; *p* < 0.001). Employed participants (OR = 1.5; 95% CI: 1.0–2.1; *p* = 0.033) and women who are age 30 or older (OR = 2.2; 95% CI: 1.6–3.1; *p* < 0.001) were more likely to have ever heard of breast cancer.

### Attitude of cervical and breast cancer

Attitudes towards cervical and breast cancer were assessed separately from the 16 questions, as shown in Tables 4 and 5. From the overall attitude score, as outlined in the methods section, the majority of the women have negative attitudes towards cervical cancer (70.6%). 70.3% among those who had heard of cervical cancer categorized it as “scary”. The majority of the interviewees also preferred female health worker to conduct cervical examination (70%). Nearly three-fourths (74.8%) perceived the examinations to be positive and believed that “health care workers doing cervical examination are not rude to women”.

**Table 4:** Attitude of cervical cancer (*n* = 337)

Questions to describe attitudes related to cervical cancer	Yes	Not sure	No not
My chances of getting cervical cancer in the next few years are high.	7.1	42.4	50.5
I feel I will get cervical cancer some time during my life.	8.3	43.3	48.4
The thought of cervical cancer scares me.	70.3	4.5	25.2
Problems I would experience with cervical cancer would last a long time.	24.9	51.9	23.2
Cervical cancer would threaten a relationship with my boyfriend, husband or partner.	35.9	40.7	23.4
If I developed cervical cancer, I would not live longer than 5 years.	11.0	52.8	36.2
Having cervical exams takes too much time.	8.3	47.8	43.9
Having cervical exams is too painful.	6.8	48.4	44.8
Health care workers doing cervical exams are rude to women.	10.4	14.8	74.8
I have other problems more important than having cervical exams in my life.	6.2	32.3	61.4
I am too old to have cervical exams regularly.	4.2	40.4	55.5
There is no health centre close to my house to have cervical exams.	34.1	14.5	51.3
If there is cancer development in my destiny, having cervical exams will not prevent it.	19.0	37.7	43.3
I prefer a female health worker to conduct cervical exams.	70.0	3.9	26.1
I will never have cervical exams if I have to pay for it.	11.0	27.9	61.1
I would be ashamed to lie on a gynecologic examination table and show my private parts to have a cervical exam.	11.9	34.7	53.4

**Table 5:** Attitude of breast cancer (*n* = 503)

Questions to describe attitudes related to breast cancer	Yes	Not sure	No
My chances of getting breast cancer in the next few years are high.	10.9	34.0	55.1
I feel I will get breast cancer some time during my life.	10.9	43.5	45.5
The thought of breast cancer scares me.	63.6	12.9	23.5
Problems I would experience with breast cancer would last a long time.	26.8	43.1	30.0
Breast cancer would threaten a relationship with my boyfriend, husband or partner.	37.8	29.4	32.8
If I developed breast cancer, I would not live longer than 5 years.	12.5	48.5	39.0
Having breast exams takes too much time.	6.4	46.3	47.3
Having breast exams is too painful.	5.4	45.3	49.3
Health care workers doing breast exams are rude to women.	11.1	15.7	73.2
I have other problems more important than having breast exams in my life.	6.6	21.7	71.8
I am too old to have breast exams regularly.	5.0	25.8	69.2
There is no health centre close to my house to have breast exams.	33.6	10.3	56.1
If there is cancer development in my destiny, having breast exams will not prevent it.	19.7	27.2	53.1
I prefer a female health worker to conduct breast exams.	65.8	2.0	32.2
I will never have breast exams if I have to pay for it.	14.1	19.7	66.2
It is embarrassing to have my breasts examined.	17.5	23.3	59.2

The majority of the participants (65.8%) preferred a female health worker to conduct breast examination. Over 73% of participants perceived the examinations to be positive and believed that “health care workers doing breast examination are not rude to women”. Over a third (37.8%) responded that “breast cancer would threaten a relationship with her husband, boyfriend or partner”. From the overall attitude score, the majority of the women were found to have negative attitudes towards breast cancer (67.4%).

There was no association between women who had ever undergone a cervical cancer screening examination and their level of education. Among women with primary and higher level of education 3.5% had ever undergone a cervical cancer screening examination compared to 3% among women with below primary level of education (OR = 1.2; 95% CI: 0.3–5.0; *p* < 0.782). However women with primary and higher level of education were 6 times more likely to had ever undergone a breast screening examination (OR = 6.0; 95% CI: 1.7–32.4; *p* = 0.001).

## Discussion

This is the first study employing a validated questionnaire to investigate the knowledge and attitudes relating to cervical and breast cancers in Ethiopia, a country with alarmingly high cancer rates. The results from this baseline study highlight the great lack of knowledge about both types of cancer. Women with primary and higher levels of education were more likely to have heard of cervical and breast cancers. These findings are consistent with other studies conducted in Ethiopia [5, 8]. Those who had heard of cervical and breast cancers largely obtained their information from family or friends, and from radio or television.

This study indicates that factors contributing to the high morbidity and mortality rates of cervical and breast cancer in Ethiopia include a general lack of awareness, ineffective screening programmes overshadowing by other health priorities (such as AIDS, TB, malaria) and insufficient attention paid to women’s health. Less than half of the women in this study were aware of cervical cancer as a disease, and only 63% of participants were aware of breast cancer despite these being the leading cancers in the country. Patients living in poor, rural communities, especially in low-income countries, often seek medical attention when cancer and metastatic diseases are at advanced stages [22]. This may also true for Ethiopian women, as regular cervical and breast cancer screenings are reported as rare in this sample. Late diagnosis leads to poor prognosis and is a pressing issue in oncology [23]. Therefore, it is necessary to address this health care issue by increasing cervical and breast cancer awareness through education and screenings.

Of those participants in this study who had heard of cervical cancer, half of this group was aware that screening was possible as a form of early detection. Despite this knowledge, less than 5% of women had ever been screened. This finding aligns with results from a study conducted in three teaching hospitals in Addis Ababa, where only 6.5% of all participants had ever undergone a Pap smear examination for cervical cancer screening [3]. Reasons cited in that study for not screening included a belief that they were not at risk for developing cervical cancer, fear of pain during the procedure as well as the fear of being diagnosed with cancer and dying shortly thereafter. Women’s knowledge about the causes and risk factors for cervical cancer was poor. Many held misconceptions about the disease and its progression. These findings are in contrast to this current study where attitudes to cervical cancer indicated a more positive attitude: respondents disagreed that cervical exams took up too much time, were painful or that staff were rude, however concerns regarding distance to health center or effect on relationships were more marked.

Similar attitudes were described concerning breast cancer. Although two-thirds of women had heard of breast cancer, only one-fifth of this group and 13.4% of the total sampled population were aware of the concept of screening. This means that even in a population of women who know about cancer, they are unaware that cancer is not inevitable in their lives and detection at a late stage can be avoided. Only 1.4% of eligible women with knowledge of breast cancer (1% of the total eligible women) had ever undergone screening. This study’s finding that a large proportion of the population remains unaware of cancer as a disease, and its consequences is an important reminder that the expansion of education and awareness is imperative for cancer control in this region.

Of the women who were aware of breast cancer, more than two-thirds had negative attitudes towards the disease. This is unsurprising, given the overabundance of late-stage, disfiguration and increased mortality seen in similar populations. Fear of breast cancer diagnosis itself can be a barrier to care, but fears of treatments has also been shown to contribute, often at increased levels, as can fears around transport and financial barriers [24]. Conversely, cancer education can also help reduce fears of the disease itself, and also help improve screening uptake, where it is available [25].

In this study, family and friends were found to be the most important source of cancer-related information, followed by media and healthcare facilities. More than half of the patients obtained health information through traditional media such as TV and radio. It should also be noted also that any formal education (primary and above) led to knowledge about breast cancer being three times more likely. These findings are important in informing methods of education and health awareness to be tailored to this population.

Methods of education which harness relational resources include peer-education, which has been used in breast screening elsewhere [26] and awareness via TV and radio, including the integration of health awareness themes into including popular television and radio dramas, with direction to healthcare facilities as part of the information. This latter strategy has been carried out for cervical cancer in other African countries to mixed results [27, 28], but remains a potentially important method of health promotion in rural low-educated communities. In considering more methods of information transfer, awareness about cancer should be made available either through audio and visual means. The difference in awareness between those with and without education highlights that improvement of all education standards, with access to basic education continues to be an important tool in public health and, in this case, cancer control.

There is an urgent necessity to inform Ethiopian women about cervical and breast cancer screenings. Efforts to promote knowledge on risk factors of female cancers should reach all women, as well as men, and provide health education and community-based interventions. Such efforts could promote a positive attitude towards treatment options, outcomes and survivorship in female cancers and to improve practices could help overcome poor awareness. It is important, however, for these to be considered in conjunction with barriers of access and availability of acceptable screening services. In this survey, a majority of women found it more favourable to be examined by a woman, and these attitudes should be considered when determining the most effective methods of screening roll-out.

In designing such programmes it is also important to note that, if the majority of the population is young or not yet of reproductive age, the burden of disease will only increase as the population ages. Therefore the importance of improving awareness of the disease, availablilty and success of screening, and early diagnostics will only increase as the younger population ages also. In this study, women under 30 years of age were less likely to have heard of breast and cervical screening, than older women. This is an opportunity to educate and inform the younger population of the importance of screenings and perhaps influence behavior change at a younger age. With a younger, more social media literate population, new models of mHealth (mobile health) technologies to access health screening and education enabled through smart phones can also be considered, as mobile phone technology continues to grow exponentially through the African continent.

Most countries in sub-Saharan Africa, including Ethiopia, do not include routine HPV vaccination in the national prevention strategy for cervical cancer and other HPV-realted diseases. Despite the challenges of implementing such a programme as part of a school health programme, there is a compelling case to be made for the introduction of HPV vaccination for adolescents aged 9 to 14 years, both male and female, as an efffective primary prevention strategy before sexual exposure to the virus [29–31].

## Conclusion

This study aimed to determine the levels of knowledge and attitudes relating to breast and cervical cancer in a rural Ethiopian female population. It found that the overall knowledge of risk factors for breast cancer and cervical cancer among women was low. Lack of cancer awareness, and lack of education in general are the most potent barriers to access and care, and should be addressed through multi-faceted strategies using peer-education, mass media and other community-based interventions. Additional research to further understand and assess the effectiveness of different strategies to increase knowledge and improve attitudes is needed.
